# 2706. Effectiveness and Safety of Shingrix Vaccination in Solid Organ Transplant Recipients at a Large Urban Transplant Center

**DOI:** 10.1093/ofid/ofad500.2317

**Published:** 2023-11-27

**Authors:** Megan Halkett, Michael Ison

**Affiliations:** Northwestern Feinberg School of Medicine, Chicago, Illinois; Respiratory Diseases Branch, DMID/NIAID/NIH, Derwood, MD

## Abstract

**Background:**

The varicella zoster virus (VZV) recombinant subunit vaccine, Shingrix, was found to be safe in solid organ transplant recipients (SOTR) and is now approved and recommended for all immunocompromised patients. Given limited efficacy data, we preferred a single center retrospective study to demonstrate safety and effectiveness of the Shingrix vaccine in solid organ transplant recipients.

**Methods:**

After IRB approval, clinical data from all SOTR at Northwestern Memorial Hospital (NMH) who received the Shingrix vaccine after their transplant, between January 1, 2017 and March 1, 2022 was collected from our Enterprise Data Warehouse (EDW). Extracted data includes age, race, ethnicity, date of transplant, organ transplanted, and date(s) of vaccine administration(s). Clinical outcomes were assessed by diagnosis of acute allograft rejection and/or HZ made post-vaccine administration. Descriptive statistics were utilized. This project is supported by the IDSA G.E.R.M. program.

**Results:**

309 of 338 eligible SOTR were included in the analysis of this study (characteristics and reasons for exclusion in Table 1). Only 1 (0.32%) patient experienced a new episode of acute allograft rejection within 3 months of Shingrix administration. 18/309 (5.8%) were diagnosed with HZ after vaccination with Shingrix. This represents a reduction in VZV reactivation events compared to the 9.1% incidence found in the 2021 meta-analysis of HZ in the general solid organ transplant population [Kwon, *Transpl Infect Dis.*, 2021].

**Table 1.**
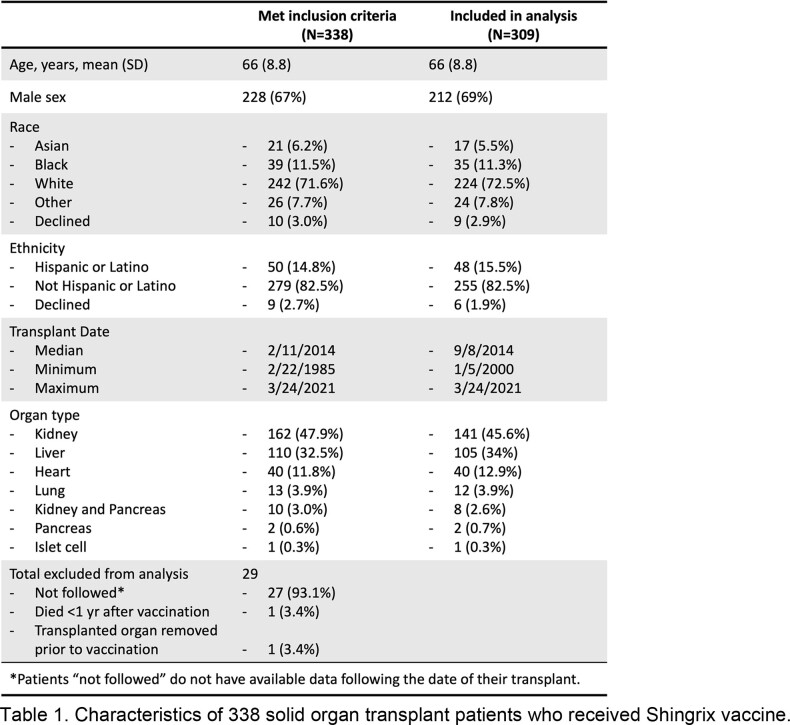
Characteristics of 338 solid organ transplant patients who received Shingrix vaccine.

**Conclusion:**

The Shingrix vaccine, when administered to SOTR post-transplant, is safe and likely effective in reducing the rate of herpes zoster. This data supports the current recommendation to vaccinate all SOTR with Shingrix. Larger, multi-center data are needed to inform the true efficacy of the vaccine in SOTRs.

**Disclosures:**

**All Authors**: No reported disclosures

